# Supplementary material for: Chronic kidney disease in Ecuador: An epidemiological and health system analysis of an emerging public health crisis
Source: PLoS One. 2022 Mar 16;17(3):e0265395. doi: 10.1371/journal.pone.0265395 (PMC8926192; doi:10.1371/journal.pone.0265395)
Supplement: S1 Table — Data are provided as numbers or percentages as indicated. (DOCX) [file pone.0265395.s001.docx]

### S1 Table. IESS Patients on Dialysis, 2015–2018.

Data are provided as numbers or percentages as indicated.

| *Year* | | ***2015*** | ***2016*** | ***2017*** | ***2018*** |
| --- | --- | --- | --- | --- | --- |
| *Total Dialysis Visits* | | 64,129 | 73,553 | 81,171 | 89,565 |
| *Type of Visit* | *Hemodialysis* | 61,265  95.5% | 70,238  95.5% | 77,807  95.9% | 86,007  96.0% |
|  | *Peritoneal Dialysis* | 2,864  4.5% | 3,315  4.5% | 3,364  4.1% | 3,558  4.0% |
| *Mean Visits per Patient* | | 9.1 | 9.3 | 9.3 | 9.3 |
| *Patients* | | 7,013 | 7,922 | 8,722 | 9,641 |
| *Sex* | *Male* | 4,488  64.0% | 5,701  64.0% | 5,573  63.9% | 6,174  64.0% |
|  | *Female* | 2,525  36.0% | 2,221  36.0% | 3,149  37.1% | 3,467  36.0% |
| *Ages* | *0-10* | 1,532  21.8% | 1,493  18.8% | 1,452  16.6% | 1,356  14.1% |
|  | *11-20* | 74  1.1% | 101  1.3% | 113  1.3% | 115  1.2% |
|  | *21-30* | 188  2.7% | 247  3.1% | 266  3.0% | 302  3.1% |
|  | *31-40* | 356  5.1% | 392  4.9% | 474  5.4% | 489  5.1% |
|  | *41-50* | 563  8.0% | 648  8.2% | 705  8.1% | 810  8.4% |
|  | *51-60* | 1,136  16.2% | 1,383  17.5% | 1,513  17.3% | 1,678  17.4% |
|  | *61-70* | 1,376  19.6% | 1,661  21.0% | 1,989  22.8% | 2,324  28.1% |
|  | *71-80* | 799  11.4% | 992  12.5% | 1,189  13.6% | 1,470  15.2% |
|  | *81+* | 250  3.6% | 347  4.4% | 395  4.5% | 514  5.3% |
|  | *Missing* | 739  10.5% | 658  8.3% | 626  7.2% | 583  6.0% |
