# Supplementary material for: Chronic kidney disease in Ecuador: An epidemiological and health system analysis of an emerging public health crisis
Source: PLoS One. 2022 Mar 16;17(3):e0265395. doi: 10.1371/journal.pone.0265395 (PMC8926192; doi:10.1371/journal.pone.0265395)
Supplement: S2 Table — Data are provided as numbers or percentages as indicated. (DOCX) [file pone.0265395.s002.docx]

### S2 Table. IESS Patients with CKD, 2015–2018.

Data are provided as numbers or percentages as indicated.

| *Year* | | ***2015*** | ***2016*** | ***2017*** | ***2018*** |
| --- | --- | --- | --- | --- | --- |
| *Patients* | | 14,757 | 18,917 | 23,926 | 29,418 |
| *Sex* | *Male* | 9,609  65.1% | 12,247  64.7% | 15,666  65.5% | 19,451  66.1% |
|  | *Female* | 5,148  34.9% | 6,670  35.3% | 8,260  34.5% | 9,967  33.9% |
| *Ages* | *0-10* | 726  4.8% | 716  3.8% | 696  2.9% | 867  2.9% |
|  | *11-20* | 116  2.1% | 152  0.8% | 207  0.9% | 222  0.8% |
|  | *21-30* | 303  4.2% | 357  1.9% | 384  1.6% | 469  1.6% |
|  | *31-40* | 625  7.3% | 662  3.5% | 817  3.4% | 909  3.1% |
|  | *41-50* | 1,073  17.4% | 1,283  6.8% | 1,591  6.6% | 1,868  6.3% |
|  | *51-60* | 2,561  24.4% | 3,097  16.4% | 3,868  16.2% | 4,555  15.5% |
|  | *61-70* | 3,595  21.4% | 4,824  25.5% | 6,402  26.8% | 7,,973  27.1% |
|  | *71-80* | 1,864  12.6% | 4,211  22.3% | 5,533  23.1% | 6941  23.6% |
|  | *81+* | 743  5.0% | 2,690  14.2% | 3,476  14.5% | 4,400  15.0% |
|  | *Missing* | 739  10.5% | 925  4.9% | 952  4.0% | 1,214  4.1% |
