# Supplementary material for: Chronic kidney disease in Ecuador: An epidemiological and health system analysis of an emerging public health crisis
Source: PLoS One. 2022 Mar 16;17(3):e0265395. doi: 10.1371/journal.pone.0265395 (PMC8926192; doi:10.1371/journal.pone.0265395)
Supplement: S3 Table — Data are provided as numbers or percentages as indicated. (DOCX) [file pone.0265395.s003.docx]

### S3 Table. MSP Patients with CKD, 2014–2018.

Data are provided as numbers or percentages as indicated.

| *Year* | | ***2014*** | ***2015*** | ***2016*** | ***2017*** | ***2018*** |
| --- | --- | --- | --- | --- | --- | --- |
| *Patients* | | 14,525 | 17,161 | 20,458 | 21,920 | 30,117 |
| *Sex* | *Male* | 7,233  49.8% | 8,735  50.9% | 10,507  51.4% | 11,207  51.1% | 15,784  52.4% |
|  | *Female* | 7,292  50.2% | 8,425  49.1% | 9937  48.6% | 10,689  48.8% | 14,326  47.6% |
|  | *Intersex* | 0  0.0% | 1  0.0% | 14  0.1% | 17  0.1% | 7  0.0% |
| *Ages* | *0-10* | 119  0.8% | 136  0.8% | 108  0.5% | 116  0.5% | 206  0.7% |
|  | *11-20* | 360  2.5% | 418  2.4% | 452  2.2% | 242  1.9% | 521  1.7% |
|  | *21-30* | 761  5.2% | 827  4.8% | 983  4.8% | 1,032  4.7% | 1,266  4.2% |
|  | *31-40* | 965  6.6% | 1,143  6.7% | 1,295  6.3% | 1,279  5.8% | 1,581  5.2% |
|  | *41-50* | 1,847  12.7% | 2083  12.1% | 2,292  11.2% | 2,416  11.0% | 3,032  10.1% |
|  | *51-60* | 3,192  22.0% | 3,606  21.0% | 4,425  21.6% | 4,582  20.9% | 6,271  20.8% |
|  | *61-70* | 3,428  23.6% | 4,066  23.8% | 5,067  24.8% | 5,576  25.4% | 7,646  25.4% |
|  | *71-80* | 2521  17.4% | 3,008  17.5% | 3,665  17.9% | 3,988  18.2% | 5,830  19.4% |
|  | *81+* | 1,323  9.1% | 1,863  10.9% | 2,146  10.5% | 2,498  11.4% | 3,764  12.5% |
|  | *Missing* | 9  0.1% | 0  0.0% | 25  0.1% | 9  0.0% | 0  0.0% |
