# Supplementary material for: Chronic kidney disease in Ecuador: An epidemiological and health system analysis of an emerging public health crisis
Source: PLoS One. 2022 Mar 16;17(3):e0265395. doi: 10.1371/journal.pone.0265395 (PMC8926192; doi:10.1371/journal.pone.0265395)
Supplement: S4 Table — (DOCX) [file pone.0265395.s004.docx]

### S4 Table. Estimated cost of dialysis to the Ecuadorian public health system.

|  | *Monthly cost per patient* (44) | *Annual cost per patient* | *Number of patients in 2018* | *Total estimated annual cost in 2018* |
| --- | --- | --- | --- | --- |
| Hemodialysis | US$1,456 | US$17,472 | 16,837 | US$294,176,064 |
| Peritoneal | US$1,245 | US$14,940 | 647 | US$9,666,180 |
| TOTAL |  | | 17,484 | US$303,842,244 |
