# Supplementary material for: Chronic kidney disease in Ecuador: An epidemiological and health system analysis of an emerging public health crisis
Source: PLoS One. 2022 Mar 16;17(3):e0265395. doi: 10.1371/journal.pone.0265395 (PMC8926192; doi:10.1371/journal.pone.0265395)
Supplement: S5 Table — (DOCX) [file pone.0265395.s005.docx]

### S5 Table. Characteristics of Interview Participants.

| **Category** | | **Total sample (n=73)** |
| --- | --- | --- |
| **Province** | Guayas | 23 (31.5%) |
|  | Pichincha | 17 (23.3%) |
|  | Manabí | 10 (13.7) |
|  | Los Ríos | 13 (17.8%) |
|  | Imbabura | 4 (5.5%) |
|  | Cañar | 4 (5.5%) |
|  | Other | 2 (2.7%) |
| **Gender** | Male | 46 (63%) |
|  | Female | 27 (37%) |
| **Type of facility** | IESS | 31 (42.5%) |
|  | Private | 27 (37%) |
|  | MSP | 13 (17.8%) |
|  | Non-profit | 2 (2.7%) |
| **Role(s)^1^** | Clinical nephrologist | 60 (41.7%) |
|  | University professor | 3 (2.1%) |
|  | Transplant program | 2 (1.4%) |
|  | Dialysis services | 34 (23.6%) |
|  | Medical director | 32 (22.2%) |
|  | General physician/internist | 12 (8.3%) |
|  | Resident doctor | 1 (0.7%) |
| **Years of experience** | 0-3 | 8 (11%) |
|  | 4-10 | 20 (27.4%) |
|  | 10+ years | 24 (32.9%) |
|  | N/A | 21 (28.8%) |
| **Nephrology training conducted outside Ecuador** | Yes | 27 (45%) |
|  | No | 7 (11.7%) |
|  | N/A | 26 (43.3%) |
| **Average number of patients seen per week^2^** | 0-20 | 9 (12.3%) |
|  | 20-40 | 15 (20.5%) |
|  | 40-80 | 16 (21.9%) |
|  | 80+ | 14 (19.2) |
|  | Not applicable or available | 19 (26%) |
| ^1^Multiple roles permitted per participant  ^2^Monthly number of patients was divided by four when necessary  IESS=Instituto Ecuatoriano de Seguridad Social, MSP=Ministerio de Salud Pública | | |
