# Supplementary material for: Chronic kidney disease in Ecuador: An epidemiological and health system analysis of an emerging public health crisis
Source: PLoS One. 2022 Mar 16;17(3):e0265395. doi: 10.1371/journal.pone.0265395 (PMC8926192; doi:10.1371/journal.pone.0265395)

### S1 Figure: Comorbidities among IESS Patients with CKD, 2015—2018

Comorbidity data were available for IESS patients with CKD who had been hospitalized in each year. The total number of comorbidities for each patient are in the plot.


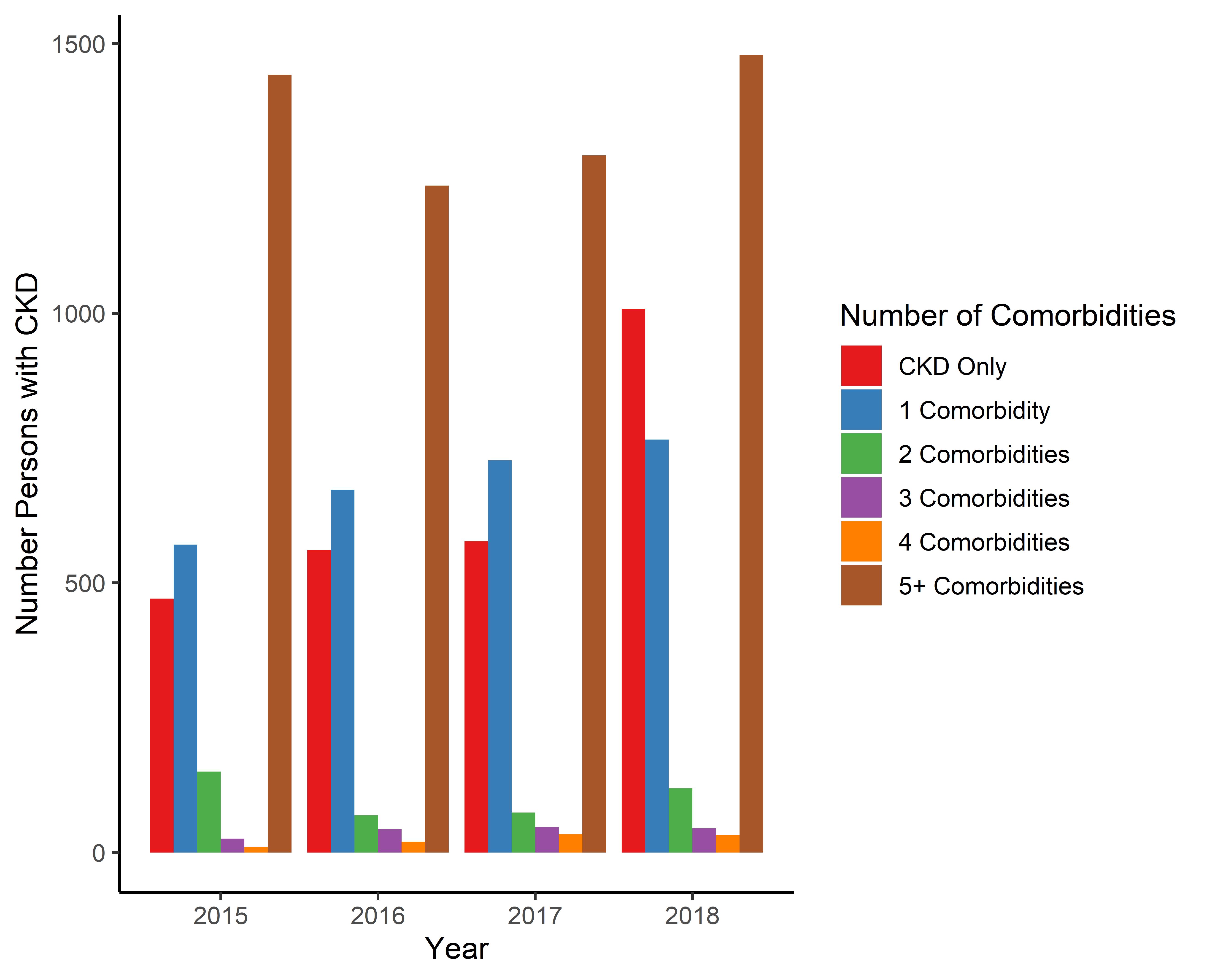

Supplement: S1 Fig — Comorbidity data were available for IESS patients with CKD who had been hospitalized in each year. The total number of comorbidities for each patient are in the plot. (DOCX) [file pone.0265395.s006.docx]
