# Supplementary material for: Chronic kidney disease in Ecuador: An epidemiological and health system analysis of an emerging public health crisis
Source: PLoS One. 2022 Mar 16;17(3):e0265395. doi: 10.1371/journal.pone.0265395 (PMC8926192; doi:10.1371/journal.pone.0265395)

### S2 Figure. Patient Travel to IESS Dialysis Clinics, 2015-2018.

Data include all dialysis service visits from 2015-2018 for patients in the IESS system, which covers 17 provinces out of 24. The location of their initial referral was compared to visits for dialysis services and whether dialysis was provided in the same province as the referral (green), a neighboring province (blue), or from farther away (red). Patients from Los Ríos, Chimborazo, and Loja had to travel most frequently to another province for dialysis.


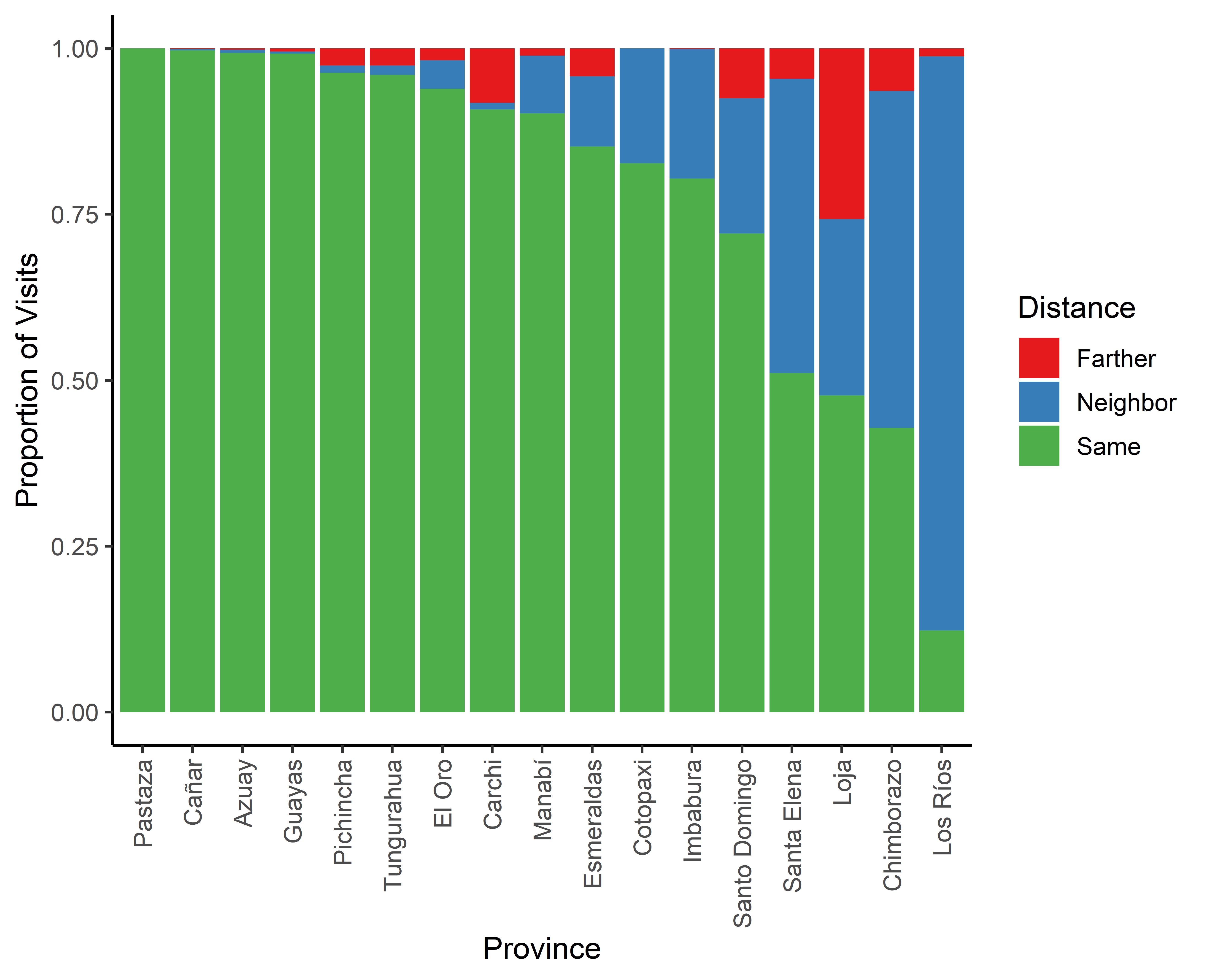

Supplement: S2 Fig — Data include all dialysis service visits from 2015–2018 for patients in the IESS system, which covers 17 provinces out of 24. The location of their initial referral was compared to visits for dialysis services and whether dialysis was provided in the same province as the referral (green), a neighboring province (blue), or from farther away (red). Patients from Los Ríos, Chimborazo, and Loja had to travel most frequently to another province for dialysis. (DOCX) [file pone.0265395.s007.docx]
