# Supplementary material for: Chronic kidney disease in Ecuador: An epidemiological and health system analysis of an emerging public health crisis
Source: PLoS One. 2022 Mar 16;17(3):e0265395. doi: 10.1371/journal.pone.0265395 (PMC8926192; doi:10.1371/journal.pone.0265395)

### S3 Figure. Patient Travel for MSP Services, 2014–2018.

Data include all CKD-related visits from 2014–2018 for patients in the MSP system, which covers 23 provinces out of 24 (Galápagos province lacks dialysis services). The location of patient residence was compared to visits for CKD-related visits and whether these visits were the same province as the patient’s residence (green), a neighboring province (blue), or from farther away (red). Patients from Bolívar and Carchi in the Andean highlands, and Zamora Chinchipe and Orellana in the Amazon region, had to travel most frequently to another province for CKD-related service.


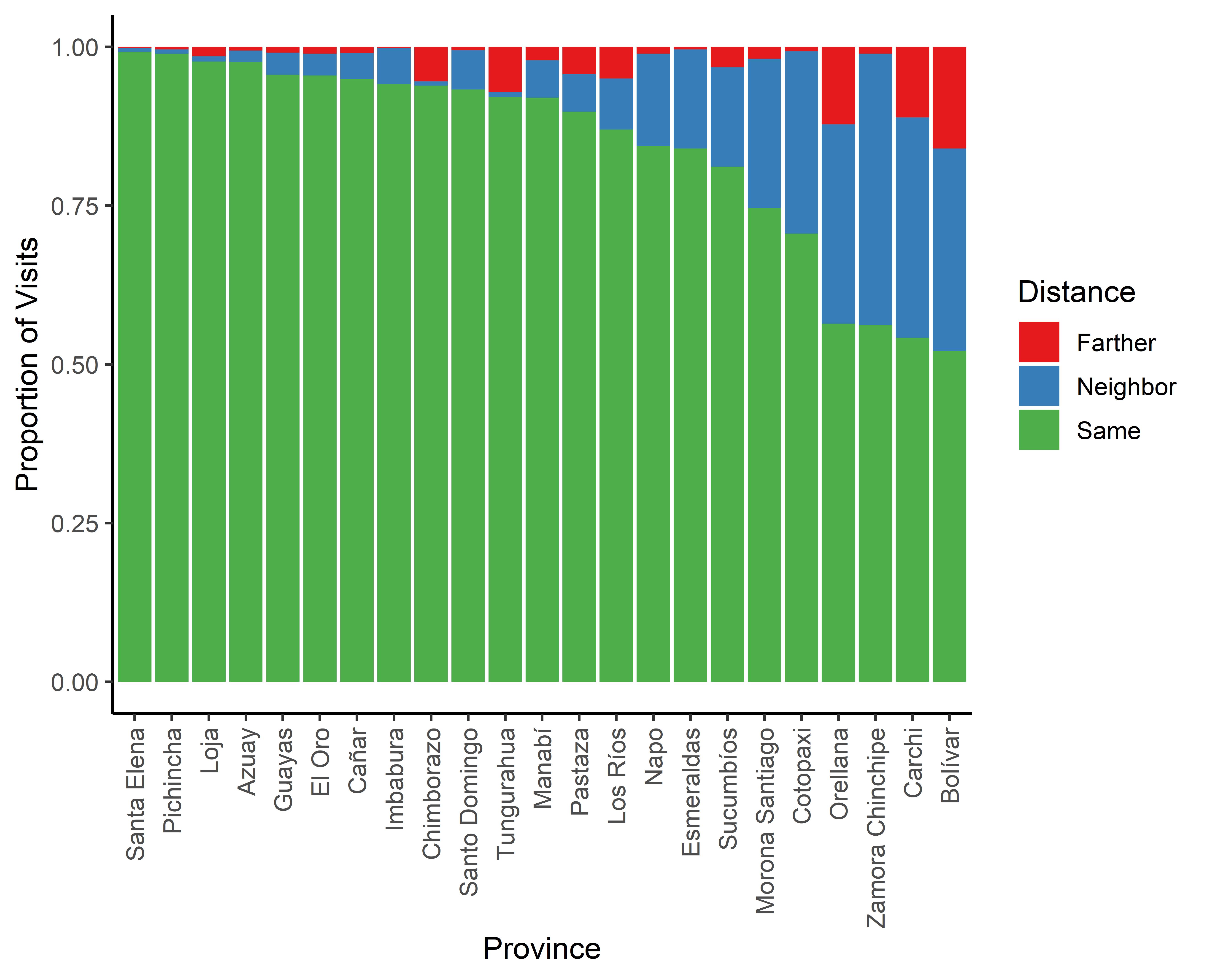

Supplement: S3 Fig — Data include all CKD-related visits from 2014–2018 for patients in the MSP system, which covers 23 provinces out of 24 (Galápagos province lacks dialysis services). The location of patient residence was compared to visits for CKD-related visits and whether these visits were the same province as the patient’s residence (green), a neighboring province (blue), or from farther away (red). Patients from Bolívar and Carchi in the Andean highlands, and Zamora Chinchipe and Orellana in the Amazon region, had to travel most frequently to another province for CKD-related service. (DOCX) [file pone.0265395.s008.docx]
