# Supplementary material for: Chronic kidney disease in Ecuador: An epidemiological and health system analysis of an emerging public health crisis
Source: PLoS One. 2022 Mar 16;17(3):e0265395. doi: 10.1371/journal.pone.0265395 (PMC8926192; doi:10.1371/journal.pone.0265395)
Supplement: S4 Fig — SOURCES: Statistical Registry of Health Resources and Activities [46]; Government of Ecuador Official Register Nº 428, Supplement, January 30, 2015; Ministry of Health Technical Guidelines 2014 [Norma técnica Subsistema de referencia, derivación, contrareferencia, referencia inversa y transferencia del Sistema Nacional de Salud]; Licensed kidney health specialized centers 2019 [Centros especializados en salud renal con licencias emitidas]; Interviews. (DOCX) [file pone.0265395.s009.docx]

### S4 Figure. Ecuadorian Health Services Network from the Perspective of an End-Stage CKD Patient.


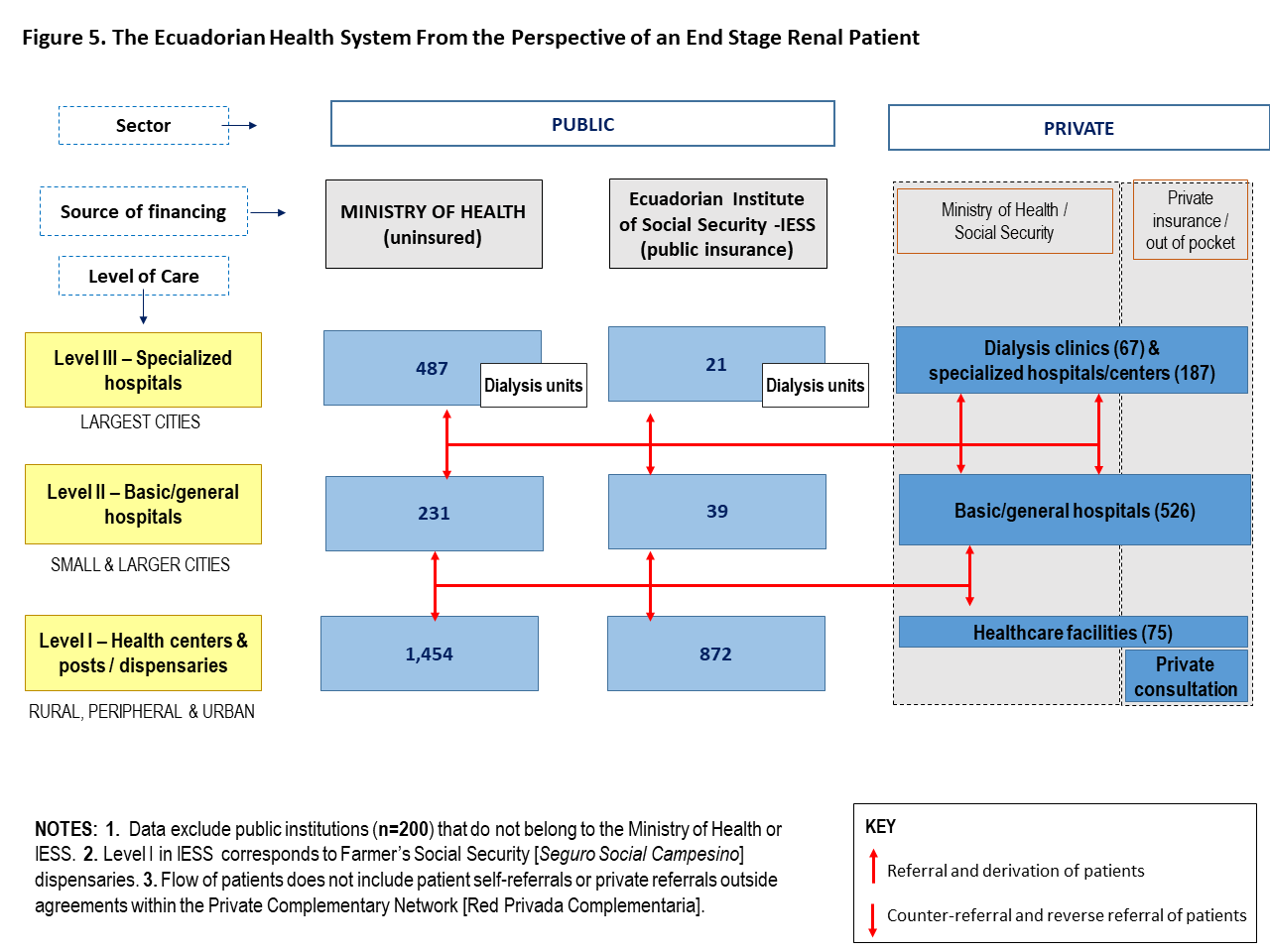


| SOURCES: Statistical Registry of Health Resources and Activities (39); Government of Ecuador Official Register Nº 428, Supplement, January 30, 2015; Ministry of Health Technical Guidelines 2014 [*Norma técnica Subsistema de referencia, derivación, contrareferencia, referencia inversa y transferencia del Sistema Nacional de Salud*]; Licensed kidney health specialized centers 2019 [*Centros especializados en salud renal con licencias emitidas*]; Interviews. |
| --- |
