# Supplementary material for: Chronic kidney disease in Ecuador: An epidemiological and health system analysis of an emerging public health crisis
Source: PLoS One. 2022 Mar 16;17(3):e0265395. doi: 10.1371/journal.pone.0265395 (PMC8926192; doi:10.1371/journal.pone.0265395)
Supplement: S1 Text — (DOCX) [file pone.0265395.s010.docx]

### S1 Text. Key Informant Interview Questions in English and Spanish.

**English Version**

**1. Can you tell me about your professional background and current position?**

Probe:

- How long have you been in practice?
- How many kidney disease patients do you see a week, on average?
- How many of these patients are on dialysis?
- Describe your place of work?

**2. What are the common causes of kidney disease in your practice?**

Probe:

- Where do most of your kidney disease patients come from (urban, rural, coast, Andes)?
  - Why do you think most of them come from this area(s)?
- Do you think kidney disease has increased in prevalence in your area over the last 10-20 years?
  - If yes, why? Explain.

**3. Do you see patients with CKDu / Meso-american nephropathy?** CKDu is understood to be permanent kidney dysfunction/failure in a young male (usually under 45 years of age) with no diabetes, high blood pressure or any other clear cause for kidney failure. There may be other members of his family, usually other young men, with the same problem.

Probe:

- How many patients do you see per week on average?
- Do your CKDu patients share any common socio-demographic factors, like gender, age, profession, socioeconomic status, place of residence, ethnicity, or something else? Explain.
  - What about mining and agricultural labor in particular?
- What type of patients bears the greatest burden of disease?
- Would you say that CKDu is predominately a disease of the poor?
- How does CKDu burden patients and their families?
- From a patient profile standpoint, how are CKDu patients different from other kidney disease patients?

**4. What are the common presentations of CKDu that you see in your clinic?**

Probe:

- How do you differentiate CKDu from other kidney diseases?
- What clinical criteria would you use to diagnose CKDu?
  - Young adult (18-40yrs)
  - Male?
  - Proteinuria?
  - Low eGFR / Low kidney function
  - Low serum potassium
  - Anything else?
- Is there any ambiguity/uncertainty in this process? How do you manage this uncertainty?
- Do your patients with kidney disease have access to prior nephrology care (pre-dialysis) before coming to you?
- Do you receive patients from other providers, or send patients to other providers?

**5. According to your understanding, what are the key factors that increase the risk of CKDu?**

Probe:

- Occupation?
- Genetic factors?
- Heat stress? Climate change
- Pesticide exposure and heavy metals in ground/drinking water
- NSAID use?
- Other factors?

**6. Do you think CKDu has increased in prevalence over the last 10-20 years in your area?**

Probe:

- Why or why not?
- How significant of a public health issue is CKDu in Ecuador?
- Do you think CKDu is different in Ecuador compared to other countries? If yes, why?
- Do you think CKDu is going to increase more in the future? Why or why not?

**7. Are you aware of any research on CKDu, or efforts to improve diagnosis, treatment and care for patients?**

Probe:

- Who? When? Where?
- Do you report cases of CKDu to the Ministry of Health? Is there any surveillance system in place?

**8. According to your understanding, what do your patients and the wider community think are the causes of CKDu?**

Probe:

- Do they connect it with pesticide exposure?
- Mining and heavy metals in ground/drinking water?
- Specific occupational hazards?
- Genetic factors?
- Heat stress? Climate change?

**9. Can you tell me about your CKDu patients and how you treat and care for them?**

Probe

- What treatments do you use and how effective are they?
  - Angiotension-converting enzyme inhibitors
  - Spironolactone
  - Potassium supplementation
  - Dialysis
  - Encourage increased fluid intake
  - Renal biopsy
  - Salt restricted diet
  - Other?
- What clinical outcomes have you seen as far?
- What has worked and not worked?
- What other problems have you encountered in attempting to care for CKDu patients?
- Do you have many patients that are lost to follow-up? Deaths?

**10. At what stage of disease do you diagnose CKDu the most?**

Probe:

- For example, end-stage renal disease – Needing dialysis
- For example, advanced kidney disease – requiring getting the patient ready for dialysis (CKD stage 4-5)
- For example, early kidney disease (CKD stage1 to 3)

**11. What barriers to early detection have you experienced with your patients?**

Probe:

- Patient’s lack of access to health care
- Lack of lab tests
- Employers’ reluctance to admit that there is an increased risk of CKDu in their patients
- Non-availability of a national database for kidney diseases
- Lack of population screening program
- Stigma
- Socio-cultural issues around health seeking behavior

**12. What barriers to treatment and care have you experienced with your CKDu patients?**

Probe:

- Patient’s lack of access to health care
- Lack of medication
- Poor health seeking behavior
- Socio-cultural issues

**13. Do you believe that there is enough attention being given to CKDu in Ecuador?**

Probe:

- Do you feel that CDKu is attracting enough attention? Why or why not?
- Are there any health education campaigns focused on kidney disease?
  - If yes, tell me about them. What was good about them and what was not so good?
- What needs to be done to raise the visibility of kidney disease and CKDu in particular?
- What resources would help you diagnose and manage kidney disease and CKDu better, to have better patient outcomes?
  - Point of care tests (blood and/or urine tests)
  - Greater community awareness?
  - Greater awareness of the problem among other health staff? (referral problems)
  - Community screening programs?

**14.** This has been a very interesting interview. You have been so helpful; I really appreciate the time you’ve taken to talk with me today. Do you have any questions for me? Is there anything else you’d like to share?

**Spanish version**

**1. ¿Puede contarme sobre su trayectoria profesional y cargo actual?**

Indague:

- ¿Cuánto tiempo ha trabajado como profesional médico?
- ¿Cuántos pacientes con enfermedad renal atiende a la semana en promedio?
- ¿Cuántos de estos pacientes se hacen diálisis?
- Describa por favor su lugar de trabajo.

**2. ¿Cuáles son las causas comunes de enfermedad renal en su consulta?**

Indague:

- ¿De dónde viene la mayoría de sus pacientes con enfermedad renal (urbano / rural; región del país)?
  - ¿Por qué cree que la mayoría viene de estas áreas?

**3. ¿Usted atiende pacientes con ERC de origen desconocido / Nefropatía Mesoamericana?** La ERC de origen desconocido es la disfunción o falla renal permanente en hombres jóvenes (usualmente menores a 45 años de edad) sin diabetes, presión arterial alta o cualquier otra causa clara para insuficiencia renal. Puede haber otros miembros en la familia, usualmente otros hombres jóvenes, con el mismo problema.

Indague:

- ¿Cuántos pacientes atiende semanalmente en promedio?
- ¿Sus pacientes con ERC de origen desconocido comparten características sociodemográficas o factores de riesgo como género, edad, profesión, estatus socioeconómico, lugar de residencia, etnicidad, o algo más?
  - Y, por ejemplo, ¿trabajo en minería o trabajo agrícola en particular?
- ¿Qué tipo de pacientes llevan la mayor carga de la enfermedad?
- ¿Usted diría que la ERC de origen desconocido es predominantemente una enfermedad de la pobreza?
- ¿Cómo afecta la ERC de origen desconocido a pacientes y sus familias?
- Tomando en cuenta los perfiles de los pacientes, ¿cómo son los pacientes de ERC de origen desconocido diferentes a otros pacientes de enfermedad renal?

**4. ¿Cuáles son las manifestaciones comunes de ERC de origen desconocido que usted ve en su práctica?**

Indague:

- ¿Cómo diferencia usted ERC de origen desconocido de otra enfermedad renal?
- ¿Qué criterios clínicos usaría usted para diagnosticar ERC de origen desconocido?
  - ¿Adulto joven (18-40 años)
  - ¿Hombre?
  - ¿Proteinuria?
  - Bajo eGFR / Baja función renal
  - Potasio sérico bajo
  - ¿Algo más?
- ¿Hay alguna ambigüedad / incertidumbre en este proceso? ¿Cómo maneja esta incertidumbre?
- ¿Sus pacientes con enfermedad renal tienen acceso a cuidado nefrológico previo (pre-diálisis) antes de ir donde usted?
- ¿Usted recibe pacientes de otros proveedores, o envía pacientes a otros proveedores?

**5. De acuerdo con lo que usted conoce, ¿cuáles son los factores clave que incrementan la ERC de origen desconocido?**

Indague:

- ¿Ocupación?
- ¿Factores genéticos?
- ¿Estrés por calor? (Cambio climático)
- Exposición a pesticidas y metals pesados en agua subterránea / segura
- ¿Uso de antiinflamatorios no esteroideos?
- ¿Otros factores?

**6. ¿Cree que la prevalencia de ERC de origen desconocido ha incrementado los pasados 10 a 20 años en su área?**

Indague:

- ¿Por qué o por qué no?
- ¿Cuán significativa es la ERC de origen desconocido como problema de salud pública en Ecuador?
- ¿Cree que la ERC de origen desconocido es diferente en Ecuador en comparación con otros países? Si es así, ¿por qué?
- ¿Cree que la ERC de origen desconocido va a incrementar o incrementar aún más en el futuro? ¿Por qué o por qué no?

**7. ¿Conoce usted de alguna investigación en ERC de origen desconocido o algún esfuerzo para mejorar el diagnóstico, tratamiento o cuidado de los pacientes?**

Indague:

- ¿Quién? ¿Cuándo? ¿Dónde?
- D¿Usted reporta los casos de ERC de origen desconocido al Ministerio de Salud Pública? ¿Hay algún sistema de vigilancia que esté funcionando?

**8. De acuerdo con lo que conoce, ¿qué es lo que sus pacientes y la comunidad más amplia consideran que son las causas de ERC de origen desconocido?**

Indague:

- ¿Lo conectan con exposición a pesticidas?
- ¿Minería y metals pesados en agua subterránea / segura?
- ¿Riesgos ocupacionales específicos?
- ¿Factores genéticos?
- ¿Estrés por calor? ¿Cambio climático?

**9. ¿Me puede contar de sus pacientes con ERC de origen desconocido y como los trata y cuida?**

Indague:

- ¿Qué tratamientos usa y cuán efectivos son?
  - Inhibidores de la enzima convertidora de angiotensina
  - Espironolactona
  - Suplementos de potasio
  - Diálisis
  - Promueve incremento en la ingesta de líquidos
  - Biopsia renal
  - Dieta restringida en sal
  - ¿Otro?
- ¿Qué resultados clínicos ha visto hasta el momento?
- ¿Qué ha funcionado y qué no ha funcionado?
- ¿Qué otros problemas ha encontrado en su cuidado de pacientes ERC de origen desconocido?
- ¿Cuántos pacientes ha perdido en el seguimiento? ¿Cuántos han muerto?

**10. ¿En qué etapa de la enfermedad usted más diagnostica ERC de origen desconocido?**

Indague:

- Por ejemplo, enfermedad renal en etapa terminal – En necesidad de diálisis
- Por ejemplo, enfermedad renal avanzada – requiring getting the patient ready for dialysis (ERC estadio 4-5)
- Por ejemplo, enfermedad renal temprana (ERC estadio1 a 3)

**11. ¿Qué barreras existen para la detección temprana que usted haya experimentado con sus pacientes?**

Indague:

- Falta de acceso de pacientes a atención en salud
- Falta de pruebas de laboratorio
- Reticencia de sus empleadores a admitir que hay un incremento de riesgo de ERC de origen desconocido en sus pacientes
- Base de datos nacional de enfermedad renal no está disponibles
- Falta de programa de tamizaje poblacional
- Estigmatización
- Factores socioculturales relacionados con comportamientos en favor de la búsqueda de salud

**12. ¿Qué barreras hacia el tratamiento y cuidado ha experimentado usted con sus pacientes con ERC de origen desconocido?**

Indague:

- Falta de acceso de pacientes a atención en salud
- Falta de medicación
- Cmportamientos poco favorables para la búsqueda de salud
- Asuntos socioculturales

**13. ¿Usted considera que se ha dado suficiente atención a la ERC de origen desconocido en Ecuador?**

Indague:

- ¿Considera que la ERC de origen desconocido atrae suficiente atención? ¿Por qué o por qué no?
- ¿Hay campañas de educación en salud que se centren en enfermedad renal?
  - Si han habido, por favor comparta lo que conoce. ¿Qué fue bueno sobre ellas y que no fue tan bueno?
- ¿Qué se debe hacer para incrementar la visibilidad de enfermedad renal y de ERC de origen desconocido en particular?
- ¿Qué recursos ayudarían a diagnosticar y manejar enfermedad renal y ERC de origen desconocido, para obtener mejores resultados con los pacientes?
  - ¿Pruebas en el punto de atención (sangre/orina)?
  - ¿Mayor sensibilización en la comunidad?
  - ¿Mayor sensibilización sobre el problema en el resto del personal de salud? (problemas al momento de referir)
  - ¿Programas de tamizaje comunitario?

**14.** Esta ha sido una entrevista muy interesante. Usted ha sido de gran ayuda; realmente le agradezco por el tiempo que se tomó para hablar conmigo. ¿Tiene alguna pregunta para mí? ¿Hay algo más que le gustaría compartir?
